# Effect of Topical Corticosteroid Treatment on microRNA Expression in Infants with Atopic Dermatitis

**DOI:** 10.1016/j.xjidi.2025.100388

**Published:** 2025-06-10

**Authors:** Janna Nousbeck, Maeve A. McAleer, Elaine M. Kenny, Alan D. Irvine

**Affiliations:** 1 Clinical Medicine, Trinity College Dublin, Dublin, Ireland; 2 Paediatric Dermatology, Children’s Health Ireland at Crumlin, Dublin, Ireland; 3 TrinSeq, Department of Psychiatry, Trinity Translational Medicine Institute, Trinity College Dublin, St James’s Hospital, Dublin, Ireland

**Keywords:** Atopic dermatitis, Biomarkers, Corticosteroids, miRNA, Therapeutics

## Abstract

MicroRNAs (miRNAs) have been implicated in a variety of disorders. Although studies have examined miRNA in pediatric atopic dermatitis (AD), the impact of topical corticosteroid (TCS) therapy on miRNA expression in pediatric AD has not been investigated. We sought to investigate the effects of 6 weeks of TCS therapy on miRNA expression in infants with AD. Small RNA sequencing and real-time RT-qPCR were performed to identify differentially expressed miRNAs in PBMCs of infants with AD after TCS treatment; HTG EdgeSeq was used to identify differentially expressed miRNAs in plasma. Kyoto Encyclopedia of Genes and Genomes pathway enrichment analysis was conducted using a list of experimentally verified miRNA targets sourced from the DIANA-TarBase and miRTarBase databases. Five miRNAs were differentially expressed in circulating PBMCs after TCS treatment (miR-143-3p, miR-27a-5p, miR-126-3p, miR-451a, and miR-223-3p); 12 miRNAs were differentially expressed in plasma. These miRNAs have regulatory functions crucial for regulating cell growth and survival, vascular adhesion, angiogenesis, skin barrier integrity, stress and nervous system processes, immune responses, inflammation, and T helper 17 cell differentiation. TCS treatment led to a distinct miRNA expression profile in peripheral blood, providing insights into how this treatment impacts disease mechanisms in childhood AD.

## Introduction

Atopic dermatitis (AD) is the most common chronic inflammatory skin disease of early childhood, affecting up to 25% of children and 7–10% of adults (Weidinger et al, 2018). The pathogenesis of AD is complex and multifactorial, involving a strong genetic predisposition, epidermal barrier dysfunction, alterations in the skin microbiome, immune dysregulation, and activation of the neuroimmune system—all of which contribute significantly to disease development. However, the overall pathological mechanism is still not fully understood (Langan et al, 2020).

MicroRNAs (miRNAs) are small noncoding RNA molecules—typically 21–24 nucleotides in length—that regulate gene expression at the post-transcriptional level by inhibiting translation or facilitating the degradation of target mRNAs, usually through sequence-specific binding to the 3' untranslated regions of their targets (Kuersten and Goodwin, 2003). miRNAs play a crucial role in regulating essential biological processes, such as cell cycle progression, apoptosis, differentiation, metabolism, and the maintenance of immune homeostasis and normal physiological functions (Saliminejad et al, 2019; Sharma et al, 2022). Most miRNAs are found within the intracellular environment; however, their presence in the extracellular environment, including blood, saliva, and urine, has also been reported (Turchinovich and Cho, 2014). A growing number of recent studies demonstrate the potential of miRNAs as biomarkers for pathogenic conditions, modulators of drug resistance, and therapeutic tools for medical interventions across diverse human health conditions (Hanna et al, 2019; Heneghan et al, 2010).

Given that AD begins in infancy, identification of early life mechanisms and responses to first-line treatments is essential. To date, most of the studies on AD miRNA expression were primarily confined to adult skin tissue (Rebane et al, 2014; Sonkoly et al, 2010). Recently, a distinct miRNA signature was shown in the peripheral blood of infants with AD, highlighting the systemic effects of the disease (Nousbeck et al, 2021). In this study, we sought to examine the effects of 6 weeks of topical corticosteroid (TCS) therapy on miRNA expression in infants with AD.

## Results

### Study patients and disease severity

**Figure 1:**
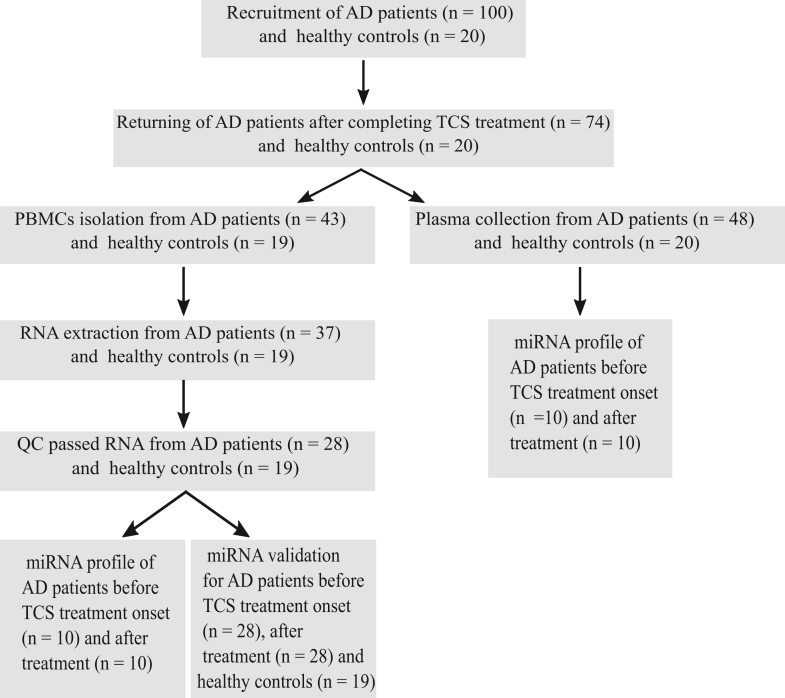
**Overall framework of study design.** AD, atopic dermatitis; miRNA, microRNA; TCS, topical corticosteroid.

One hundred infants with moderate or severe AD and 20 healthy control infants were initially recruited (McAleer et al, 2021). From this cohort, 74 patients returned for examination after 6 weeks of TCS therapy. PBMCs were isolated from 43 patients and 19 controls. RNA samples extracted from PBMCs of 28 patients (collected both before and after TCS therapy) and 19 controls that met quality-control criteria were included in further analysis. In addition, 10 plasma samples were collected from patients with AD both before and after TCS therapy and used for miRNA analysis. The use of samples is presented in a schematic flow chart (Figure 1). Clinical and demographic features are summarized in Table 1, Table 2.

**Table 1:** Clinical and Demographic Characteristics of Study Participants Examined for miRNA Expression in PBMCs

Characteristics	Patients with AD		Healthy Subjects
Total	28		19
Sex			
Male	19		12
Female	9		7
Age, mo			
Median	6.5		7
Range	3–10		0–12.3
Age of AD onset, wk			
Median	8^1^		—
Range	2-20		—
SCORAD	T0	T6	
Average	49.4	17.9	—
Range	23.4–91.3	0–79.6	—
Comorbidities			
Asthma			
Present	1		—
Absent	25		—
Unknown	2		—
Food Allergies			
Present	10		—
Absent	16		—
Unknown	2		—
Allergic rhinitis			
Present	—		—
Absent	26		—
Unknown	2		—

Abbreviations: AD, atopic dermatitis; miRNA, microRNA; SCORAD, SCORing Atopic Dermatitis; TCS, topical corticosteroid.

T0 denotes before TCS treatment; T6 denotes after TCS treatment.

1 Missing 1 age of onset.

**Table 2:** Clinical and Demographic Characteristics of the 10 Participants Examined for Plasma Levels of miRNAs

Characteristics	Patients with AD T0	Patients with AD T6
Total	10	
Sex		
Male	8	
Female	2	
Age, mo		
Median	6.5	
Range	4–10	
Age of AD onset, wk		
Median	8	
Range	2–20	
SCORAD		
Average	48.3	16.95
Range	31.8–65	0–44.7
Comorbidities		
Asthma		
Present	—	
Absent	9	
Unknown	1	
Food allergies		
Present	3	
Absent	7	
Unknown	—	
Allergic rhinitis		
Present	—	
Absent	9	
Unknown	1	

Abbreviations: AD, atopic dermatitis; miRNA, microRNA; SCORAD, SCORing Atopic Dermatitis; TCS, topical corticosteroid.

T0 denotes before TCS treatment; T6 denotes after TCS treatment.

**Figure 2:**
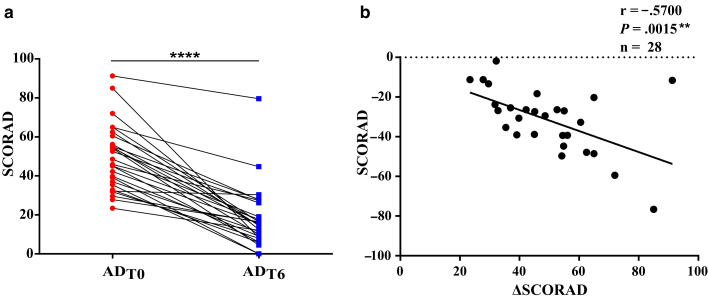
**(a) Disease severity as assessed by SCORAD in infants with AD at baseline (ADT0) and after 6 weeks of TCS therapy (ADT6).** Data between ADT0 and ADT6 groups were compared using Wilcoxon signed-rank test. **(b)** Spearman’s rank correlation analysis comparing baseline disease severity (SCORAD) with changes in SCORAD (ΔSCORAD). ∗∗*P* < .01 and ∗∗∗∗*P* < .001. AD, atopic dermatitis; SCORAD, SCORing Atopic Dermatitis; TCS, topical corticosteroid.

Most children showed significant improvement in disease severity after TCS therapy, as assessed by SCORing Atopic Dermatitis (SCORAD) (Table 1), with only 1 child showing no improvement (Figure 2a). Spearman's rank correlation analysis revealed a strong association between baseline disease severity (SCORAD) and the decrease in disease severity (ΔSCORAD) (r = −0.57, *P* < .01) (Figure 2b), indicating that children with greater baseline disease severity tend to experience a more pronounced decrease in disease severity after TCS treatment.

### miRNA expression in PBMCs of children with AD after 6 weeks of TCS treatment

**Figure 3:**
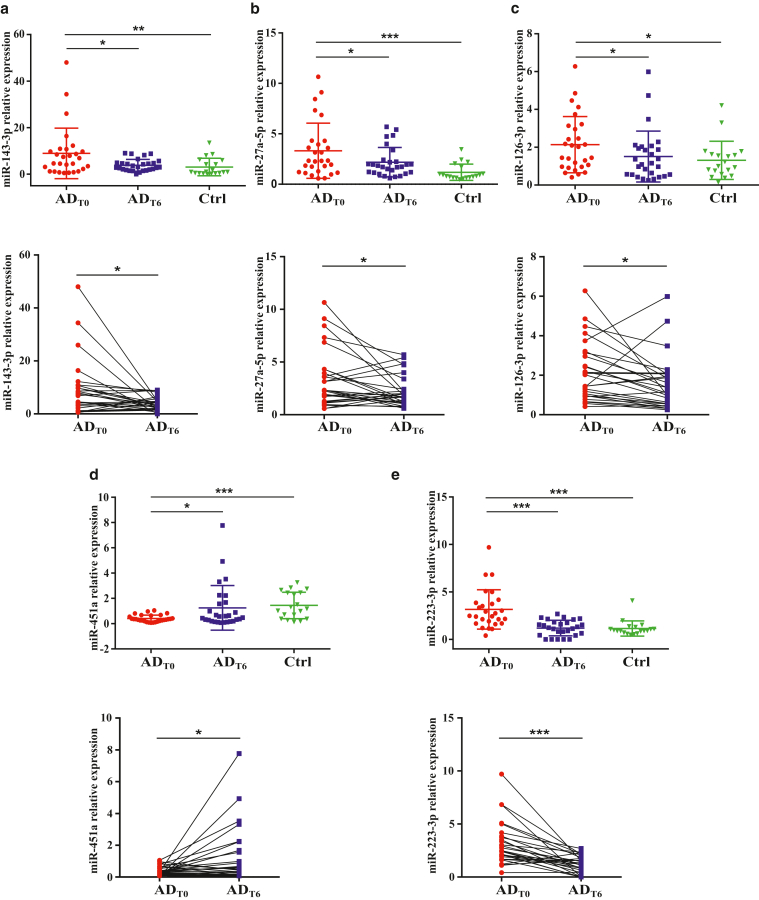
**Validation of differentially expressed miRNAs in PBMCs of infants with AD after TCS treatment.** RT-qPCR analyses of potential miRNA biomarkers in paediatric AD after TCS therapy: relative expression of (**a**) miR-143-3p, (**b**) miR-27a-5p, (**c**) miR-126-3p, (**d**) miR-451a, and (**e**) miR-223-3p in PBMCs of infants with AD at baseline (AD_T0_) (n = 28) and after 6 weeks of topical corticosteroid therapy (AD_T6_) (n = 28) and heathy controls (denoted as Ctrl) (n = 19). Fold change was calculated by 2^-ΔΔCT^ method. The normalized expression data are shown as the means ± SD. Data between groups were compared using Mann–Whitney *U* test for unpaired samples (AD_T0_ vs Ctrl) and Wilcoxon signed-rank test for paired samples (AD_T0_ vs AD_T6_). FDR-corrected *P*-values are ∗FDR < 0.05, ∗∗FDR < 0.01, and ∗∗∗FDR < 0.001. AD, atopic dermatitis; FDR, false discovery rate; miRNA, microRNA; TCS, topical corticosteroid.

Recently, it was demonstrated that infants with AD exhibit a distinct peripheral blood miRNA signature (Nousbeck et al, 2021). To evaluate the effects of a 6-week treatment with TCSs on the miRNA expression profile in infants with AD, we initially conducted small RNA-sequencing analysis, a high-throughput, comprehensive method that profiles miRNA expression, detecting both known and unknown miRNAs and allowing for the precise identification of miRNA sequences (Pritchard et al, 2012; Wang et al, 2009). We compared the miRNA profiles of PBMCs between patients at baseline (n = 10) and after 6-week TCS therapy (n = 10). More than 500 miRNAs that passed the low-count filter were tested for differential expression analysis. Using a cut off of false discovery rate (FDR) < 0.05 and log_2_ fold change (FC) ≥ 1, we found 3 significantly differentially expressed miRNAs: miR-143-3p, miR-126-3p, and miR-27a-5p (Table 3). miR-143-3p has been previously associated with inflammation (Løvendorf et al, 2014), mir-126 has been reported to be involved in T helper 2 responses (Mattes et al, 2009), and miR-27a has been shown to be involved in the regulation of inflammatory response of macrophages (Xie et al, 2014). Both miR-143-3p and miR-126-3p have been previously reported by us to be differentially expressed in infants with AD compared with those in healthy controls (Nousbeck et al, 2021). To further validate the differential expression of miR-143-3p, miR-27a-5p, and miR-126-3p, identified by RNA-sequencing analysis in this study, we performed miRNA RT-qPCR in a larger number of samples and compared the expression between 3 groups: patients with AD before treatment onset (n = 28) and after the therapy (n = 28) and healthy controls (n = 19). The levels of miR-143-3p markedly decreased after treatment (FDR = 0.0468) (Figure 3a). Similarly, the levels of miR-27a-5p significantly decreased after treatment (FDR = 0.0477), although they did not reach those of healthy participants (Figure 3b). The expression of miR-126-3p was also significantly reduced by treatment (FDR = 0.0110) (Figure 3c). As expected, all 3 miRNAs showed significant differences between healthy controls and patients prior to treatment.

**Table 3:** Differential Expression Analysis of miRNAs in PBMCs of infants with AD after TCS Treatment

miRNA	LFC	*P*-Value	FDR
hsa-miR-143-3p	−1.4385	<.001	0.0135
hsa-miR-27a-5p	−1.0337	<.001	0.0135
hsa-miR-126-3p	−1.0169	<.001	0.0497

Abbreviations: AD, atopic dermatitis; FDR, false discovery rate; LFC, log_2_ fold change; miRNA, microRNA; TCS, topical corticosteroid.

Presented are top differentially expressed miRNAs identified through small RNA-sequencing analysis of PBMCs from children with AD during their first year of life after treatment (n = 10), compared with those PBMCs from the same children before treatment onset (n = 10). Differential expression analysis was performed using package DESeq2 in R software. miRNAs with |LFC| ≥ 1 and FDR < 0.05 are shown.

miR-451a, previously identified as a candidate biomarker for pediatric patients with AD (Nousbeck et al, 2021), did not meet the low-count filter criteria for both pre and post-treatment groups in this study, hindering the validation of its differential expression after treatment through RNA-sequencing analysis. miR-223-3p, another biomarker previously found to be differentially expressed in pediatric patients with AD (Nousbeck et al, 2021) and implicated in AD pathogenesis (Jia et al, 2018), did not meet the initial cutoff criterion (FDR < 0.05) when comparing patients at baseline (n = 10) with those after 6 weeks of therapy (n = 10), despite having a significant *P*-value (< .05) (Supplementary Table S1). Although miR-451a did not meet the low-count filter, and miR-223-3p did not meet the FDR cutoff criteria in the current RNA-sequencing analysis, we proceeded to analyze their expression using miRNA RT-qPCR, which offers high sensitivity and sequence specificity, enabling precise detection of expression levels, particularly at low levels. We compared the expression levels of these miRNAs across 3 larger groups: patients with AD before treatment (n = 28), those after therapy (n = 28), and healthy controls (n = 19). As shown in Figure 3d, miR-451a levels significantly increased after treatment (FDR= 0.0448) and reached those of healthy participants. In contrast, miR-223-3p expression levels significantly decreased after treatment (FDR = 0.0005) (Figure 3e). As expected, the levels of both miRNAs, miR-451a, and miR-223-3p exhibited significant differences between healthy controls and patients prior to treatment.

In summary, by utilizing both RNA-sequencing analysis and RT-qPCR, each with its own advantages, we identified significant changes in the expression levels of 5 miRNAs—miR-143-3p, miR-27a-5p, miR-126-3p, miR-451a, and miR-223-3p—after topical treatment in pediatric patients with AD.

### Correlation between differentially expressed miRNAs, disease severity, and other clinical parameters

We then explored the relationship between differentially expressed miRNAs, disease severity, and clinical parameters in pediatric patients with AD after TCS treatment. First, we investigated whether baseline levels of differentially expressed miRNAs were correlated with disease severity, as assessed by SCORAD. Spearman’s rank correlation analysis revealed a significant positive correlation between miR-223-3p expression at baseline and SCORAD (r = 0.4, *P* < .039), whereas no significant correlations were observed for the other miRNAs (miR-143-3p, miR-27a-5p, miR-126-3p, and miR-451a). We also examined whether miRNA expression levels were associated with changes in disease severity (ΔSCORAD) but found no significant correlations between baseline miRNA expression or changes in miRNA expression and ΔSCORAD. Furthermore, Spearman’s analysis of the impact of comorbidities (food allergy, asthma, and allergic rhinitis) on miRNA expression showed no significant correlations, as indicated by high *P*-values and insignificant rho values. In addition, no significant associations were found between clinical parameters, such as disease onset and sex, and miRNA expression levels. Multivariate regression analysis also failed to identify significant predictors of miRNA expression, including disease severity and other clinical parameters and comorbidities. These findings suggest that the identified miRNAs—miR-143-3p, miR-27a-5p, miR-126-3p, miR-451a, and miR-223-3p—do not appear to be influenced by clinical parameters or changes in disease severity after treatment.

### Prediction and annotation of miRNA putative target genes

Next, we aimed to explore the disease-related targets of the 5 miRNAs found to be significantly changed in infants with AD after TCS therapy. We searched for predicted targets using online databases of experimentally verified miRNA–gene interactions: DIANA-TarBase (Skoufos et al, 2024) and miRtarbase (Huang et al, 2022). An integrative list of experimentally verified targets for each miRNA was further uploaded to functional annotation tool of DAVID (The Database for Annotation, Visualization and Integrated Discovery) database for pathway analysis (Huang da et al, 2009; Sherman et al, 2022).

**Figure 4:**
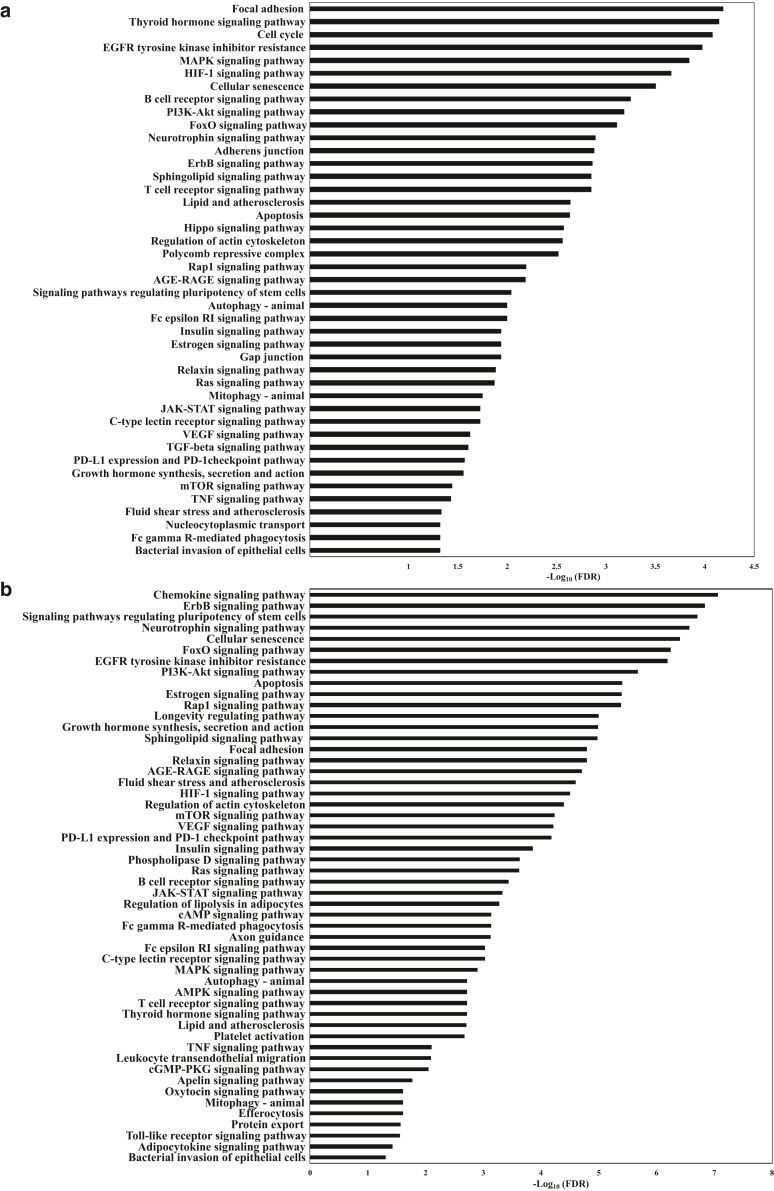

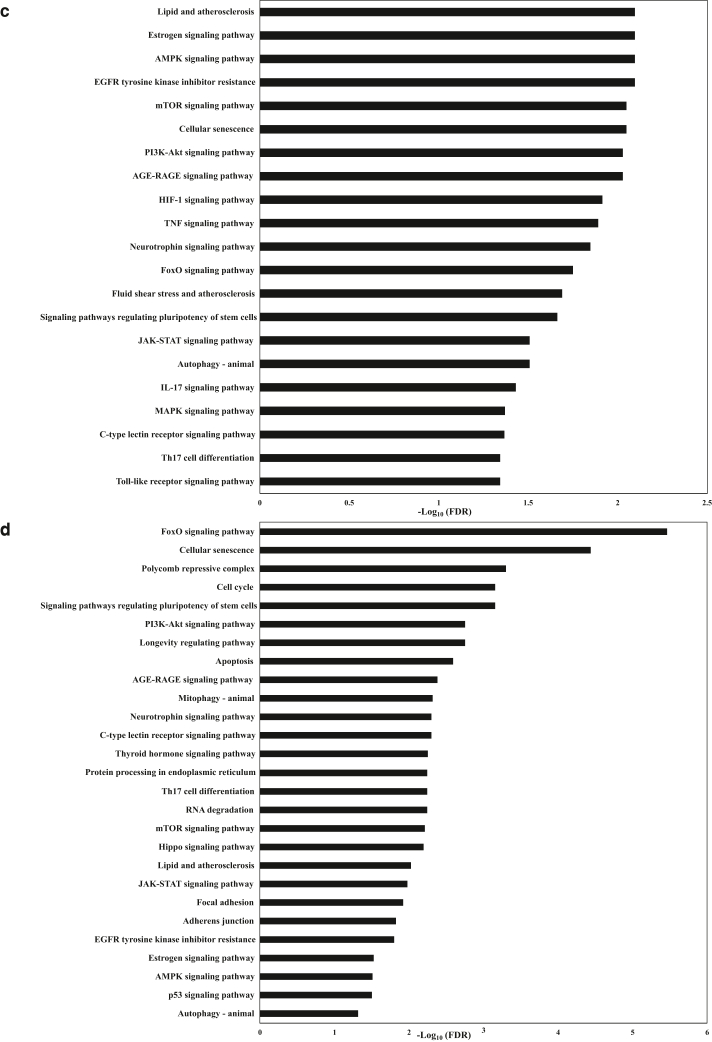
**KEGG pathway analysis of predicted targets of differentially expressed miRNAs in PBMCs of infants with AD after topical corticosteroids therapy.** Predicted targets of differentially expressed miRNAs in PBMCs of infants with AD after TCS treatment were identified using databases of experimentally verified miRNA–gene interactions—DIANA-TarBase and miRtarbase—and were analyzed through KEGG pathway enrichment analysis with the DAVID tool. Significantly enriched KEGG pathways (fold enrichment > 1.5 and FDR < 0.05) potentially related to immune system regulation, AD, or TCS treatment are shown for predicted targets of (**a**) miR-143-3p, (**b**) miR-126-3p, (**c**) miR-451a, and (**d**) miR-223-3p. Horizontal axis is the enrichment of pathways, and vertical axis is the pathway category. AD, atopic dermatitis; DAVID, Database for Annotation, Visualization and Integrated Discovery; FDR, false discovery rate; KEGG, Kyoto Encyclopedia of Genes and Genomes; miRNA, microRNA; TCS, topical corticosteroid.

We identified 33 significantly enriched gene ontology terms represented in the biological process category for miR-143-3p targets, including regulation of transcription, gene expression, regulation of cell cycle and cell proliferation, apoptotic processes, and many others. Using the DAVID tool, we obtained more than 80 significantly enriched pathways. The enriched pathways were associated with angiogenesis (VEGF signaling pathway, HIF-1 signaling pathway), cell growth and apoptosis (Ras signaling pathway, FoxO signaling pathway, Hippo signaling pathway, ErbB signaling pathway, and other cell cycle/apoptosis pathways), immune response (ie, Fc epsilon RI signaling pathway, TCR signaling pathway, TGF-β signaling pathway, Fc gamma R-mediated phagocytosis), and inflammatory response (ie, TNF signaling pathway, MAPK signaling pathway, Jak/signal transducer and activator of transcription signaling pathway) and pathways related to stress and nervous system (ie, neurotrophin signaling pathway, long-term depression, long-term potentiation, and Alzheimer’s disease pathway) (Figure 4a and Supplementary Table S2).

We discovered 25 significantly enriched gene ontology terms within the biological process category and over 100 significantly enriched pathways for miR-126-3p targets. Among the enriched pathways, we identified angiogenesis-associated pathways (VEGF signaling pathway, HIF-1 signaling pathway), cellular senescence and apoptosis, immune response pathways (ie, TCR signaling pathway, B-cell receptor signaling pathway, leukocyte transendothelial migration, mTOR signaling pathway), inflammatory response pathways (ie, TNF signaling pathway, MAPK signaling pathway, Jak/signal transducer and activator of transcription signaling pathway, toll-like receptor signaling pathway, phosphoinositide 3-kinase–protein kinase B pathway), and pathways related to stress and nervous system (ie, neurotrophin signaling pathway and Alzheimer’s disease pathway) (Figure 4b and Supplementary Table S2).

Among the 8 pathways significantly enriched for miR-27a-5p targets, we identified WNT signaling pathway and adherens junction pathway (Supplementary Table S2).

More than 40 significantly enriched pathways were identified for miR-451a targets, many of which were associated with immune system function (Figure 4c and Supplementary Table S2). These include TNF signaling pathway, IL-17 signaling pathway, T helper 17 cell differentiation, toll-like receptor signaling pathway, FoxO signaling pathway, mTOR signaling pathway, Jak–signal transducer and activator of transcription signaling pathway, MAPK signaling pathway, phosphoinositide 3-kinase–protein kinase B signaling pathway, among others.

We also identified over 20 significantly enriched gene ontology terms in the biological process category, along with >50 significantly enriched pathways for miR-223-3p targets (Figure 4d and Supplementary Table S2). These enriched pathways are involved in processes such as cell cycle regulation, cell death, and apoptosis, including the apoptosis pathway, FoxO signaling, phosphoinositide 3-kinase–protein kinase B signaling, p53 signaling, cell cycle, and cellular senescence, as well as immune response pathways such as T helper 17 cell differentiation and C-type lectin receptor signaling and nervous system pathways such as the neurotrophin signaling pathway, along with various other biological processes.

### miRNA expression in the plasma of children with AD at baseline and after treatment

In addition to PBMCs, plasma samples collected from patients at baseline and after treatment were used for HTG EdgeSeq, providing insights into the plasma miRNA profile. This platform combines a nuclease-free library preparation for targeted transcripts followed by next-generation sequencing for quantitation of the transcripts. The expression of 2083 human miRNA transcripts was measured, and miRNA counts were compared between patients at baseline (n = 10) and after 6-week therapy (n = 10). Using a cut off of FDR < 0.05 and log_2_ FC ≥ 1, we found 12 circulating miRNAs to be differentially expressed between the 2 groups (Table 4 and Supplementary Table S3). Among differentially expressed miRNAs, we identified miR-146a-5p that has been previously shown to be involved in the pathogenesis of AD (Rebane et al, 2014).

**Table 4:** Differential Expression Analysis of miRNAs in Plasma of Infants with AD after TCS Treatment

miRNA	LFC	*P*-Value	FDR
miR-1287-5p	−1.119	.001	0.037
miR-375	1.023	<.001	0.005
miR-1291	1.051	<.001	0.011
miR-146a-5p	1.164	.0013	0.04
miR-19a-3p	1.189	.0015	0.041
miR-106b-5p	1.228	<.001	0.037
miR-6741-5p	1.229	<.001	0.022
miR-19b-3p	1.373	<.001	0.011
miR-21-5p	1.392	.0016	0.041
miR-664b-5p	1.526	<.001	0.001
miR-122-5p	1.566	<.001	0.011
miR-3687	1.600	<.001	0.005

Abbreviations: AD, atopic dermatitis; FDR, false discovery rate; LFC, log_2_ fold change; miRNA, microRNA; TCS, topical corticosteroid.

Presented are top differentially expressed miRNAs obtained from HTG EdgeSeq analysis of plasma samples from children with AD at first year of life after treatment (n = 10) compared with those from the same children before treatment onset (n = 10). Differential expression analysis was performed using package DESeq2 in R software. miRNAs with |LFC| ≥ 1 and FDR < 0.05 are shown.

## Discussion

miRNAs, essential post-transcriptional regulators of gene expression, have potential as disease biomarkers and therapeutic targets, providing insights into biological pathways for diagnostic and treatment applications. Because AD typically begins in infancy, understanding early mechanisms and responses to treatments is crucial. Our recent findings show a distinct peripheral blood miRNA signature in infants with AD, emphasizing the systemic nature of the disease (Nousbeck et al, 2021). This work extends previous studies and explores the impact of 6 weeks of TCS therapy on miRNA expression in infants with AD. By examining miRNA changes before and after TCS treatment, we aimed to gain a deeper understanding of how treatment affects disease mechanisms in early childhood.

Our cohort consisted of pediatric patients with AD aged <1 year with moderate-to-severe AD. Most children demonstrated significant improvement in disease severity after TCS therapy, as assessed by SCORAD. In addition, a strong correlation between baseline disease severity (SCORAD) and the decrease in disease severity (ΔSCORAD) indicated that children with more severe baseline disease are more likely to show a greater decrease in severity after TCS treatment.

Using RNA-sequencing analysis and RT-qPCR, we identified 5 miRNAs that were differentially expressed in circulating PBMCs from children with AD after TCS treatment. These miRNAs include miR-143-3p, miR-27a-5p, miR-126-3p, miR-451a, and miR-223-3p. As expected, expression levels of all 5 miRNAs were significantly different between healthy controls and patients with AD before treatment. miR-143-3p and miR-223-3p, both myeloid specific (Cantoni et al, 2017; Lam et al, 2018), have been shown to be upregulated in PBMCs and lesional skin of patients with psoriasis (Løvendorf et al, 2014), suggesting a role in inflammatory skin diseases. miR-223 has also been implicated in the pathogenesis of AD (Jia et al, 2018). miR-27a-5p has been reported to play a significant role in regulating inflammatory processes (Wang et al, 2024) and immune response modulation, particularly influencing the balance between T helper 17 and regulatory T cells (Lu et al, 2024). miR-126 has been reported to be involved in T helper 2 responses (Mattes et al, 2009) and has shown significant differential expression in infants with AD in our previous research (Nousbeck et al, 2021). miR-451a has been shown to play a critical role in oncogenesis (Liu et al, 2016; Wang et al, 2015; Zhai et al, 2024) and erythroid lineage differentiation (Patrick et al, 2010). In addition, our previous findings demonstrated that miR-451a, either alone or in combination with miR-223 and/or miR-143, could serve as an efficient diagnostic biomarker for AD (Nousbeck et al, 2021). We wondered whether changes in miRNA expression levels after treatment were associated with clinical factors such as disease onset, sex, or disease severity or whether comorbidities (food allergy, asthma, and allergic rhinitis) influenced changes in miRNA expression. However, no significant correlations were identified. These findings suggest that neither comorbidities nor other available clinical parameters appear to affect miRNA expression changes after treatment in our pediatric patients with AD. The lack of association between changes in miRNA expression and disease severity may be due to the limited sample size available for analysis as well as the possibility that miRNA expression reflects early or systemic molecular changes that do not immediately result in visible skin improvement. Furthermore, clinical measures such as SCORAD may not fully capture the complexity of the biological processes influenced by miRNAs.

HTG EdgeSeq analysis of plasma samples revealed 12 differentially expressed miRNAs after TCS therapy. Among differentially expressed miRNAs, we identified miR-146a-5p that has been previously reported to be anti-inflammatory miRNA. Interestingly, overexpression of miR-146a inhibited proinflammatory factors in IFN-γ– and TNF-α–stimulated human primary keratinocytes (Rebane et al, 2014). Furthermore, in a mouse model of AD, miR-146a–deficient mice developed severe skin inflammation with heavy infiltrations in the dermis, strong hyperplasia, and thickening of the epidermis (Rebane et al, 2014). Although the identification of these distinct miRNAs in plasma is promising, we acknowledge that the small sample size (n = 10) presents a limitation (Kok et al, 2018). It reduces statistical power and may hinder the detection of smaller effect sizes or variations in miRNA expression, including interindividual differences. Therefore, future studies with larger patient cohorts are essential to validate these findings and provide a more comprehensive understanding of the results.

One of the questions we sought to address was the identification of biological pathways targeted by the 5 differentially expressed miRNAs in PBMCs of infants with AD after TCS therapy. To explore this, we performed Kyoto Encyclopedia of Genes and Genomes pathway enrichment analysis using a list of experimentally verified miRNA targets sourced from the DIANA-TarBase and miRTarBase databases. We found that miR-143-3p targets were significantly enriched in over 80 biological pathways, particularly those related to immune system responses, inflammation, apoptosis, cell growth regulation, angiogenesis, and stress and nervous system functions. For miR-126-3p targets, >100 significantly enriched pathways were identified, including those associated with angiogenesis, immune response, inflammatory response, cellular senescence and apoptosis, and stress and nervous system functions. Analysis of miR-27a-5p targets showed significant enrichment in WNT signaling pathway and adherens junction pathway. miR-451a targets were significantly enriched in over 40 pathways, with notable enrichment in immune system–related pathways. miR-223-3p target pathway analysis showed significant enrichment in >50 pathways, including those related to cell cycle regulation, cell death, apoptosis, and immune response pathways and nervous system functions.

The identified pathways play crucial roles in regulating cell growth and survival, maintaining tissue and organ homeostasis, and mediating immune modulation and inflammatory responses. For instance, phosphoinositide 3-kinase–protein kinase B–mTOR signaling pathway, a central regulator of tissue and organ homeostasis, plays a major role in immune-mediated dermatoses such as AD (Roy et al, 2023). Similarly, Jak–signal transducer and activator of transcription signaling pathway is critical in AD pathogenesis (Kopalli et al, 2022), with Jak inhibitors emerging as key therapeutic options (Armario-Hita et al, 2023). Hippo signaling pathway, which regulates cell proliferation, survival, and inflammation, is also implicated in AD (Jeong and Lee, 2023). In addition, HIF-1 pathway plays a crucial role in immune responses, inflammation, and skin barrier function (McGettrick and O'Neill, 2020; Wong et al, 2015). WNT and adherens junction pathways are crucial for skin conditions such as AD (Lee et al, 2022; Niessen, 2007), with adherens junctions playing a key role in maintaining the epidermal barrier (Yoshida et al, 2022). T helper 17 signaling pathway, with T helper 17 T cells expressed at higher levels in the skin of pediatric patients with AD than in adults with AD (Esaki et al, 2016), plays a role in immune responses and serves as a potential therapeutic target (Sirvent et al, 2023). Another highly relevant pathway, neurotrophin signaling pathway, contributes to neuroimmune interactions, inflammation, pruritus, and skin barrier dysfunction in AD (Steinhoff et al, 2022; Weihrauch et al, 2023).

TCSs are widely used to treat dermatological conditions owing to their strong anti-inflammatory, antimitotic, and immunosuppressive effects. They exert broad, multitargeted actions across various cellular and molecular pathways (Ahluwalia, 1998; Gabros et al, 2024). In this study, we demonstrated that 6 weeks of TCS treatment led to differential expression of several miRNAs in the peripheral blood of infants with AD, with their targets linked to immune response and inflammation, T helper 17 cell differentiation, vascular adhesion, angiogenesis, cell growth, survival, skin barrier integrity, and nervous system function. These findings reflect a wide range of effects of TCS, providing insights into how short-term TCS therapy influences mechanisms related to AD. However, to fully understand the mechanisms involved in TCS treatment for pediatric AD, it is important to explore gene expression profiles in infants after treatment. By integrating miRNA profiles with gene expression data, a more comprehensive understanding of the molecular networks can be achieved. This approach may help clarify the mechanisms contributing to both the pathogenesis of AD and the response to TCS therapy. Such insights could reveal how TCS treatment influences disease progression and systemic changes in early childhood, ultimately informing more personalized and effective treatment strategies for managing AD in infants.

In conclusion, our study demonstrated that TCS treatment in infants with AD led to a distinct miRNA expression profile in peripheral blood, highlighting a systemic effect of short-term TCS therapy and providing insights into how such treatment influences disease mechanisms in early childhood.

## Materials and Methods

### Patient recruitment, clinical assessment, and therapy

Infants aged <12 months with moderate-to-severe AD were recruited, as previously described (Nousbeck et al, 2021), on the basis of a SCORAD index ≥25 and a disease duration of at least 6 weeks. The participants were treatment naïve, apart from the use of emollients and 1% hydrocortisone cream or ointment. Age-matched healthy controls were recruited when attending Children’s Health Ireland at Crumlin, Dublin for elective procedures under general anaesthetic. Controls were included only if there was no diagnosis or history suggestive of AD, atopy, or any other inflammatory skin condition. All children were examined to ensure an absence of inflammatory skin disease. Written informed consent was obtained from the parents or legal guardians for all participants. The age of onset of AD was documented. Severity was assessed using the SCORAD scale (European Task Force on Atopic Dermatitis, 1993). Patients were treated with TCSs in line with normal clinical practice; treatment course was tailored to the child’s disease severity according to department practice. Potencies ranged from class VII to class II at a maximum of 1.5 grams daily for the 6-week duration.

### Study samples

A total of 100 infants with moderate or severe AD and 20 healthy control infants were initially recruited (McAleer et al, 2021). PBMCs were isolated from 43 patients and 19 controls. RNA samples extracted from PBMCs of 28 patients (collected both before and after TCS therapy) and 19 controls that met quality-control criteria were included in further analysis. These 28 patient and 19 control samples were also used in our previous miRNA study (28 patients at baseline and 19 controls) (Nousbeck et al, 2021) as well as in our gene expression profiling study (8 patients at baseline and 5 controls) (Nousbeck et al, 2023). In addition, 10 plasma samples were collected from patients with AD both before and after TCS therapy and used for miRNA analysis. These plasma samples obtained before TCS treatment onset were also utilized in our previous study (Nousbeck et al, 2021).

### Sample preparation, RNA extraction, and quality control

PBMCs were isolated from whole blood, as previously described (Nousbeck et al., 2021), using histopaque double-gradient density centrifugation (Sigma-Aldrich) and were cryopreserved for further analysis. Total RNA was isolated from PBMCs according to miRNeasy Mini Kit protocol (Qiagen). RNA integrity and quality were assessed using RNA 6000 Nano Lab Chips on an Agilent 2100 Bioanalyzer (Agilent Technologies). Only RNA samples with optimal RNA Integrity Number values (≥8) were selected for library preparation and sequencing. Plasma was isolated from whole blood according to standard protocols.

### Small RNA libraries preparation

Libraries for miRNA sequencing were prepared using the NEXTflex Small RNA-Seq Kit (version 3) according to the manufacturer’s instructions (Bioo Scientific), as previously described (Nousbeck et al, 2021). Briefly, 400 ng of total RNA were used for library preparation. After ligation of the RNA 3′- and the RNA 5′- adapter, total RNA samples were reverse transcribed to cDNA libraries, and unique indices were introduced during 18 cycles of PCR amplification. PCR products were size selected and purified using PAGE-based method. Final libraries were assessed for quality using Bioanalyzer 2100 (Agilent) and quantified with a Qubit fluorometer (Thermo Fisher Scientific).

### RNA-sequencing data processing and differential expression analysis

Equimolar amounts of purified libraries were pooled together, quantified, and denatured. Five percent of a PhiX control library (Illumina) was spiked into the pools. The denatured pooled libraries at the concentration of 12 pM were loaded onto the MiSeq sequencing cartridge (Illumina). Sequencing was performed as previously described (Nousbeck et al, 2021) using the Illumina MiSeq sequencing platform in single-end mode for 50 cycles, following the manufacturer’s instructions. Data were stored and managed using BaseSpace Sequence Hub (Illumina). Sequencing reads demonstrated high quality, with a mean Phred score >30 for 97% of all bases. After sequencing, raw reads were uploaded to the Galaxy research platform (https://usegalaxy.org/) to eliminate adapter sequence and random bases from the 3′ prior to alignment. Trimmed reads were subsequently filtered, aligned, and analyzed using the BaseSpace Small RNA app (version 1.0.1) (Illumina). Differential miRNA expression analysis was performed using the DESeq2 package in R software (version 3.5.2), considering an expression >20 read counts in at least 25% of the samples, a cut off of log_2_ FC ≥ 1, and a Benjamini–Hochberg corrected FDR < 0.05.

### Real-time RT-qPCR

miRNAs of interest were analyzed using real-time RT-qPCR as previously described (Nousbeck et al, 2021). Briefly, RNA isolated from PBMCs was reverse transcribed using the TaqMan Advanced MicroRNA cDNA Synthesis Kit (Applied Biosystems) that generated universal cDNA templates for all miRNAs present in the sample. Then, miRNA-specific TaqMan Advanced miRNA assays (Applied Biosystems) were used for real-time qPCR on a 7900HT Fast Real-Time PCR System (Applied Biosystems). The expression of target miRNAs in PBMCs was normalized to the mean of miR-151a-5p, miR-93-5p, and miR-103a-3p, selected as reference miRNAs for their lowest variance among sequenced miRNAs and high expression stability across the tested samples, as determined by analysis tools such as NormFinder, geNorm, and BestKeeper. Each reaction was performed in triplicate, and the results are expressed as the mean ± SD. The FC in target miRNAs relative to their expression in healthy controls was determined using the 2^-ΔΔCT^ method (Livak and Schmittgen, 2001). FC data were log_2_ transformed for correlation analysis with AD comorbidities and other clinical parameters.

### HTG EdgeSeq

Plasma samples (n = 10 for patients before treatment onset and n = 10 after the treatment) were sent to HTG EdgeSeq miRNA Whole Transcriptome Assay for targeted sequencing (HTG Molecular Diagnostics), as previously described (Nousbeck et al, 2021). Briefly, HTG Plasma Lysis Buffer was added to each of the samples. The functional DNA Nuclease Protection Probes were added to the lysed samples and hybridized to RNA. After nuclease digestion, which degrades nonhybridized DNA probes and nonhybridized RNA, and subsequent denaturation to release the DNA protection probes, each sample was tagged with molecular tags, adaptors required for cluster generation, and a unique barcode for sample identification and multiplexing. Tagged libraries were pooled in equal amounts with 5% of control PhiX and sequenced on an Illumina MiSeq sequencer. Sequence analysis was carried out using EdgeSeq Parser software (HTG Molecular Diagnostics). Differential miRNA expression analysis was performed as described earlier.

### Target genes prediction and functional enrichment

The potential targets of the differentially expressed miRNAs were identified using 2 databases of experimentally verified miRNA-gene interactions: DIANA-TarBase (Skoufos et al, 2024) and miRTarBase (release 9) (Huang et al, 2022). Both shared and unique genes were present in each database; therefore, genes from either database were included in the integrated list of verified miRNA targets. This list was then uploaded to DAVID tools (Huang da et al, 2009; Sherman et al, 2022). Using the DAVID tool, we conducted gene ontology analysis of the targets to identify biological processes, molecular functions, and pathways. Significantly enriched Kyoto Encyclopedia of Genes and Genomes pathways (fold enrichment > 1.5 and FDR < 0.05) were used for further analysis.

### Statistical analysis

Statistical analysis was performed using R software (version 3.5.2) with the package DESeq2, the Graph Pad Prism (version 6.0, GraphPad software), and the Statistical Package for the Social Sciences software (version 30.0, IBM).

Differential miRNA expression analysis on RNA-sequencing data was performed using package DESeq2 in R software (version 3.5.2). FDR, based on Benjamini–Hochberg method, was used to correct for multiple comparisons of miRNA expression levels between patients with AD at baseline and those at after 6-week treatment.

Relative expression of highly significant miRNAs identified by differential expression analysis (FDR < 0.05, log_2_ FC ≥ 1) and miRNAs of interest was analyzed by RT-qPCR using the 2^-ΔΔCT^ method against healthy controls group. Data between groups were compared using Mann–Whitney *U* test for unpaired samples (AD_T0_ [patients with AD at baseline] vs control [healthy controls]) and Wilcoxon signed-rank test for paired samples (AD_T0_ [patients with AD at baseline] vs AD_T6_ [after 6 weeks of TCS therapy]). *P*-values were calculated, and FDR correction was been applied. FDR < 0.05 was considered to indicate a statistically significant difference. FC data obtained from RT-qPCR using the 2^-ΔΔCT^ method were log_2_ transformed, and correlation analyses to evaluate the association between miRNA expression levels and AD comorbidities and clinical parameters were conducted using Spearman’s rank and multivariate regression in Statistical Package for the Social Sciences software.

## Ethics Statement

This study was approved by the Research Ethics Committee of Our Lady’s Children’s Hospital Crumlin (now Children’s Health Ireland at Crumlin). Written informed consent was obtained from the parents or legal guardians for all participants.

## Data Availability Statement

Datasets related to this article can be found at https://osf.io/yzqr8/.

## ORCIDs

Janna Nousbeck: http://orcid.org/0000-0001-6918-8855

M. A. McAleer: http://orcid.org/0000-0001-9958-6504

E. M. Kenny: http://orcid.org/0000-0003-4676-3436

Alan D. Irvine: http://orcid.org/0000-0002-9048-2044

## Conflict of Interest

The authors state no conflict of interest.

## Supplementary Material

Supplementary Table S1 Differential Expression Analysis of miRNAs in PBMCs of Infants with AD after TCS Treatment XXX Abbreviations: AD, atopic dermatitis; miRNA, microRNA; TCS, topical corticosteroid. miRNAs identified through small RNA-sequencing analysis that passed the low-count filter were subjected to differential expression analysis, comparing miRNAs from PBMCs of infants with AD after TCS treatment (n = 10) with those from the same infants before treatment (n = 10). Differential miRNA expression analysis was performed using package DESeq2 in R software (version 3.5.2).

Supplementary Table S2 KEGG Pathway Analysis of Predicted Targets of Differentially Expressed miRNAs in Infants with AD after TCS XXX Abbreviations: AD, atopic dermatitis; DAVID, Database for Annotation, Visualization and Integrated Discovery; FDR, false discovery rate; KEGG, Kyoto Encyclopedia of Genes and Genomes; miRNA, microRNA; TCS, topical corticosteroid. Predicted targets of differentially expressed miRNAs in PBMCs of infants with AD after TCS treatment were identified using databases of experimentally verified miRNA–gene interactions—DIANA-TarBase and miRtarbase—and were analyzed through KEGG pathway enrichment analysis with the DAVID tool. Significantly enriched KEGG pathways (fold enrichment > 1.5 and FDR < 0.05) are presented.

Supplementary Table S3 Differential Expression Analysis of miRNAs in Plasma of Infants with AD after TCS Treatment XXX Abbreviations: AD, atopic dermatitis; miRNA, microRNA; TCS, topical corticosteroid. miRNAs identified through HTG EdgeSeq analysis were subjected to differential expression analysis, comparing plasma samples from infants with AD after TCS treatment (n = 10) with those from the same infants before treatment (n = 10). Differential miRNA expression analysis was performed using package DESeq2 in R software.
